# CT characteristics and laboratory findings of COVID-19 pneumonia in relation to patient outcome

**DOI:** 10.1186/s43055-020-00385-x

**Published:** 2021-01-14

**Authors:** Ibrahim A. I. Mohamed, Hosam A. Hasan, Mohamed Abdel-Tawab

**Affiliations:** Department of Diagnostic Radiology, Assiut University, Assiut, 71515 Egypt

**Keywords:** COVID-19, Viral pneumonia, Ground-glass opacities, D-dimer

## Abstract

**Background:**

This study aimed to investigate the chest computed tomography (CT) characteristics and laboratory findings in patients with confirmed COVID-19 pneumonia and to evaluate their relationship with clinical outcome.

This retrospective study assessed *164* consecutive CT chests of COVID-19 patients during April 2020. The chest CT and laboratory data were analyzed. The primary endpoint was patient survival either died or survived. The relationship between CT and laboratory findings was correlated to patient outcome.

**Results:**

The study group included 164 patients (86 male, 78 women; average age, 44.3 ± 16.5 years) whose RT-PCR were positive for COVID-19. Only 120 (73.2%) patients had pulmonary manifestations. Ground glass opacities of peripheral distribution and multifocal affection were the major CT finding in COVID-19 patients. Univariate analysis revealed that CT severity score, D-dimer level, age, total leucocytic count, and absolute lymphocytic count were predictive for death.

**Conclusion:**

CT has an emerging role in the diagnosis of COVID-19 pneumonia and in assessing disease severity. CT severity score, D-dimer, total leucocytic count, and absolute lymphocytic count significantly predict patient survival.

## Background

Coronavirus disease 2019 (COVID-19) was announced as a global pandemic in March 2020 [1], with the worldwide infected cases exceeding 26 million and the deaths over 870,000 as of September 2020 [2]. In Egypt, as of 2 September 2020, the number of cases had reached 99280 and over 5400 deaths [3].

COVID-19 patients present with a wide range of symptoms ranging from being asymptomatic or with simple upper respiratory tract infection to severe acute respiratory distress syndrome (ARDS) [4]. Therefore, rapid diagnosis of the affected cases is crucial for early patient isolation and limitation of disease spread as there is no specific vaccine or curative treatment [5].

The gold-standard technique for diagnosis is reverse transcription-polymerase chain reaction (RT-PCR), with high specificity. Still, this investigation’s downside is the lack of sensitivity and the lengthy time to reach diagnosis [6]. On the other hand, computed tomography (CT) is a readily available fast technique and high sensitivity [7]. Despite having a very high sensitivity, which can be up to 97.2% [8], unfortunately, CT has a very low specificity, which can reach as little as 25% [7]. Thus, this has made CT one of the cornerstones in diagnosing COVID-19 infection [7]. Laboratory data are also playing an essential role in the detection and management of COVID-19 patients, with the lymphocytic count being one of the most critical severity indicators [9].

This study aims to assess the CT and laboratory findings of COVID-19 infected patients and correlate them with the patient outcome.

## Methods

### Patient population

The institutional review board approved this study, where 164 patients with confirmed real-time reverse transcriptase-polymerase chain reaction (RT-PCR) for COVID-19 were consecutively admitted in April 2020, and their CT chest images were reviewed. We excluded patients without a chest CT scan and unstable patients with low image quality. CT images of the patients were reviewed. The patients’ laboratory data were obtained, which included the leukocytic count, absolute lymphocytic count, hemoglobin level, platelet count, D-dimer level, and C-reactive protein.

### Image acquisition

CT chest scanning was performed for all patients using a 16-channel CT scanner (Aquilion Lightning; Toshiba Medical Systems). The scanning range included the whole chest from the thoracic inlet down to the diaphragm. All the patients were scanned without contrast media injection. The patient was in the supine position and scanned with breath holding at the end of inspiration. The scanning parameters were as following: tube voltage, 120 kV; tube current, 50 mA; rotation time, 0.5 s; and slice thickness of 5 mm.

### CT analysis

Three experienced radiologists evaluated all the CT chest examinations independently, and the discrepancies were resolved by agreement. CT images of each patient were assessed for the presence and distribution of parenchymal abnormalities, including ground-glass opacities (GGO), consolidation, multifocality, distribution (peripheral or diffuse), septal thickening, crazy paving, pulmonary nodules, pleural effusion, and mediastinal lymph nodes with short-axis > 1 cm. Also, the CT severity score was assigned for each lobe as the following: 0 for no involvement, 1 for < 5% involvement, 2 for 5–25% involvement, 3 for 25–50% involvement, 4 for 50–75%% involvement, and 5 for > 75% involvement, then multiplied by 5 to calculate the overall severity score. A mild grade is of 0–7 points, a moderate grade is of 8–16 points, and an advanced grade is of 17–25 points

### Statistical analysis

Categorical data were reported in frequencies and percentages. Continuous data were reported as mean ± standard deviation if normally distributed and in median (interquartile range) if not normally distributed. A univariate analysis yielding odds ratios with 95% confidence intervals (95% CIs) was applied to test predictors of death in COVID-19 patients. For statistical analysis, we used SPSS version 26 (SPSS Inc., Chicago, IL, USA). Statistical significance was defined as *p* < 0.05.

## Results

### Patient demographics

In this retrospective study, we included 164 patients diagnosed as COVID-19, 86 were male (52.4%), and 78 were female (47.6%); ranging in age from 1 to 85 years (mean age 44.3 ± 16.5 years). Laboratory data of the patients are shown in Table 1.

**Table 1:** Laboratory results of patients

Laboratory parameter	Normal range	Result
Total leucocytic count (× 10^3^/μL)	3.5–9.5	6.6 ± 4.0
Lymphocytes count (× 10^3^/μL)	1.1–3.2	1.69 ± 1.15
Platelets (× 10^3^/μL)	150–450	240 ± 76.8
Hemoglobin ((g/dL)	Male (13–17.5); female (12–15)	12.7 ± 1.7
D-dimer (ng/mL)	< 250	1200 ± 320
C-reactive protein (mg/L)	< 8	31.4 ± 30.1

### CT-findings

**Fig. 1:**
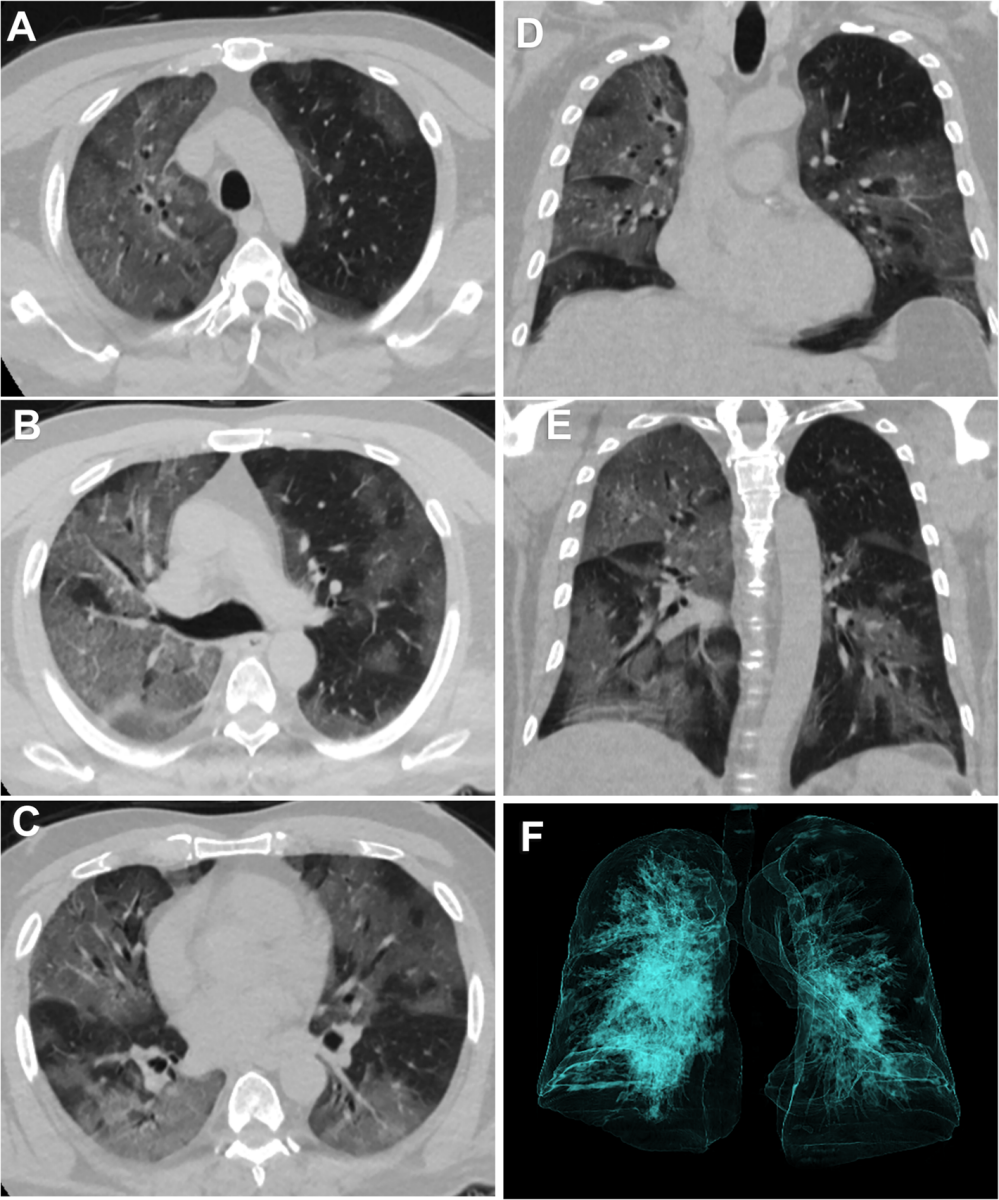
Non-contrast axial CT of the chest of a 58-year-old male patient with positive RT-PCR. Bilateral diffuse, more peripheral ground-glass opacities are seen with prominent vessels in the affected areas (**a**–**e**). 3D reconstruction volume rendering image shows pulmonary involvement as opaque non-transparent shadows (**f**). CT severity score is 16 points which corresponds to moderate involvement. The patient died on follow-up

**Fig. 2:**
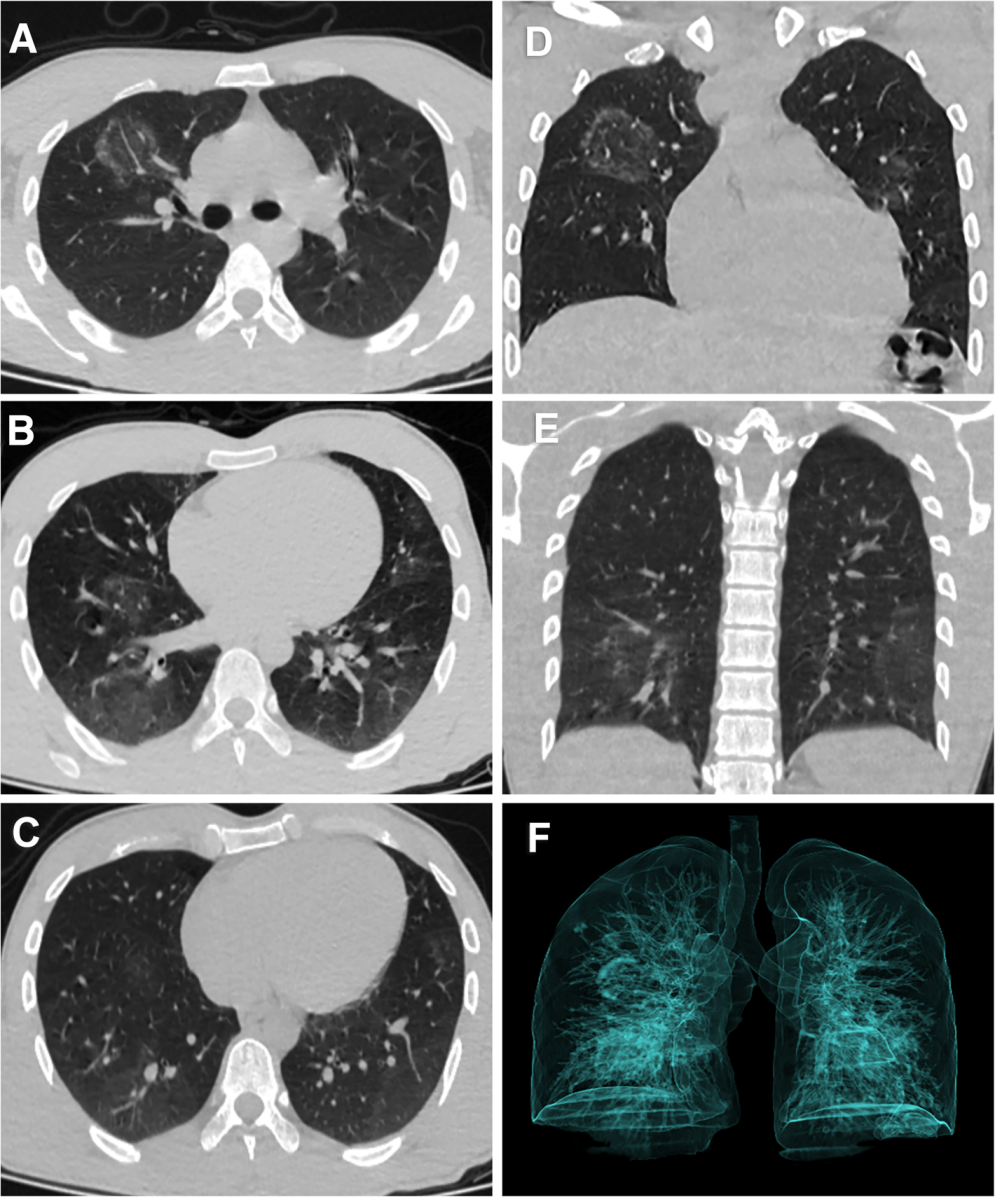
Non-contrast axial CT of the chest of a 30-year-old male patient presented with fever and dyspnea with positive RT-PCR. The right upper lobe shows a reverse halo “atoll sign” in axial and coronal cuts (**a**, **d**). Bilateral ground-glass opacities are seen at lower lobes with peripheral distribution (**b**, **c**, **e**). 3D reconstruction volume rendering image shows pulmonary involvement as opaque non-transparent shadows (**f**). CT severity score is 7 points which corresponds to mild involvement. The patient improved on follow-up

**Fig. 3:**
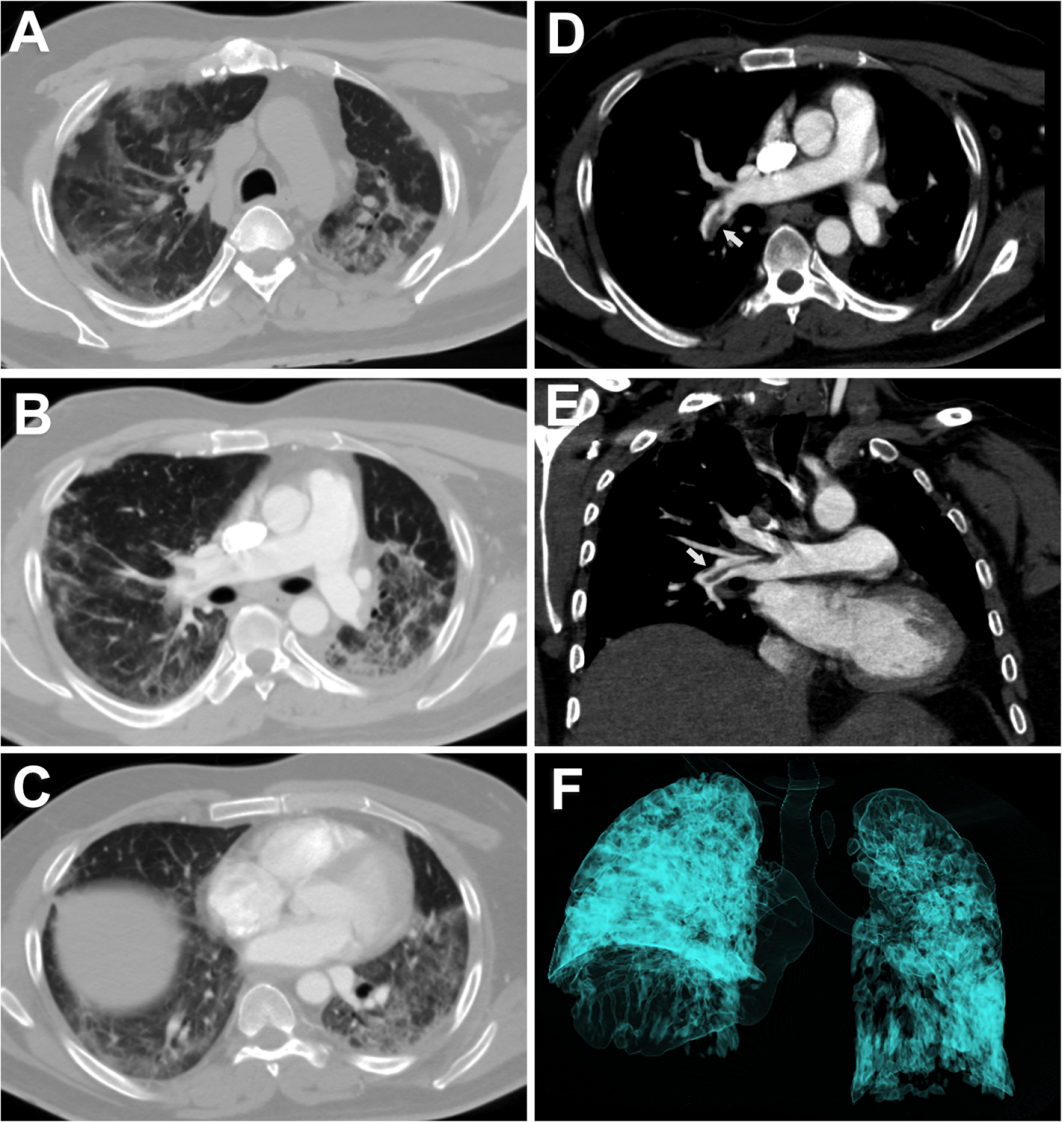
Non-contrast axial CT of the chest of a 45-year-old male patient with positive RT-PCR. Bilateral ground-glass opacities are seen of mainly peripheral distribution (**a**–**c**). The patient had a high D-dimer level. Thus, CT pulmonary angiography was done (**d**, **e**), revealing right descending pulmonary arterial thromboembolism (arrows). 3D reconstruction volume rendering image shows pulmonary involvement as opaque non-transparent shadows (**f**). CT severity score is 15 points which corresponds to moderate involvement. The patient received anticoagulant treatment and improved on follow-up

The initial chest CT studies of the 164 patients with COVID-19 showed that 44 patients had CT with no pulmonary affection (26.8%). In the affected patient group, the distribution of lesions was predominantly peripheral in 86 (71.7%) patients while it was diffuse in 34 (28.3%) patients. Multifocal affection was seen in 109 (90.8%) patients. The major CT finding observed in COVID-19 patients was GGO found in 118 (98.3%) patients. Consolidation was found in 52 patients (43.3%), while septal thickening was found in 41 patients (34.2%). The least encountered CT findings were crazy paving pattern found in 9 (7.5%) patients, reverse halo sign found in 3 (2.5%), pulmonary nodules found in 4 (3.3%), and enlarged mediastinal lymph nodes found in 6 (5%) while no patient had pleural effusion (Table 2) (Figs. 1, 2 and 3).

**Table 2:** CT findings in COVID-19 patients

	Total (***N*** = 164)	***N*** = 120 (excluding the normal cases)
GGO	118 (72%)	118 (98.3%)
Multifocal	109 (66.5%)	109 (90.8%)
Distribution		
Peripheral	86 (52.4%)	86 (71.7%)
Diffuse	34 (20.7%)	34 (28.3%)
Consolidation	52 (31.7%)	52 (43.3%)
Septal thickening	41 (25%)	41 (34.2%)
Crazy paving	9 (5.5%)	9 (7.5%)
Reverse halo	3 (1.8%)	3 (2.5%)
Nodules	4 (2.4%)	4 (3.3%)
Lymphadenopathy	6 (3.6%)	6 (5%)
Pleural effusion	0 (0%)	0 (0%)

*GGO* Ground-glass opacities

### CT severity score

The average CT severity score was 6.81; the interquartile range was 0.5–11.5. The most commonly affected parts were the lower lobes, mainly at dorsal segments. The proportion of pneumonic involvement per each lobe is shown in Table 3.

**Table 3:** The proportion of pneumonic involvement per each lobe

	RUL	RML	RLL	LUL	LLL
0% involvement	69	70	53	66	53
1–5% involvement	34	34	27	46	36
5–25% involvement	36	36	35	30	32
25–50% involvement	14	14	30	15	27
50–75% involvement	7	7	14	6	12
> 75% involvement	4	3	5	1	4

*RUL* Right upper lobe, *RML* Right middle lobe, *RLL* Right lower lobe, *LUL* Left upper lobe, *LLL* Left lower lobe

### Regression

The endpoint of the study was patient survival, where 11 patients (6.7%) died. We found that age, total leucocytic count, absolute lymphocytic count, D-dimer level, and CT-severity score could predict survival in patients with COVID-19 pneumonia (Table 4). CT severity score had the most statistically significant predictive value (*p* value = 0.000, OR = 1.327). It is followed by D-dimer level (*p* value = 0.004, OR = 1.389). Age is also a significant predictor ((*p* value = 0.018, OR = 1.060). The total leucocytic count and the absolute lymphocytic count also had a statistically significant predictive value (*p* value = 0.019 and 0.043, OR = 1.193 and 0.272, respectively).

**Table 4:** Logistic regression of predictors of death

	beta	*p* value	OR	95% CI	
				Lower	Upper
Age	0.059	**0.018**	1.060	1.010	1.113
Gender	− 0.133	0.847	0.876	0.227	3.385
Total leucocytic count	0.176	**0.019**	1.193	1.029	1.383
Lymphocyte count	− 1.302	**0.043**	0.272	0.077	0.960
Platelets	− 0.008	0.112	0.992	0.981	1.002
Hemoglobin	− 0.011	0.881	0.989	0.858	1.140
C-reactive protein	− 0.081	0.314	0.922	0.787	1.080
D-dimer	0.329	**0.004**	1.389	1.114	1.732
CT severity score	0.289	**0.000**	1.336	1.158	1.540

*Statistically significant difference (*p* < 0.05)

*OR* Odds ratio, *CI* Confidence interval

## Discussion

COVID-19 is an emerging disease which is caused by SARS-CoV-2. It causes extensive edema of the interlobular septae with lymphocytic infiltration in the pulmonary interstitium. Although the early airspace and alveolar exudate accumulation are not significant, the disease may progress dramatically [10].

CT has a cornerstone in the diagnosis of COVID-19 patients in many situations. A study by Dawoud et al. [11] recommended CT chest CT for diagnosis and follow-up of COVID-19 pneumonia. A study conducted by Ali et al. [12] who studied COVID-19-associated pneumonia in asymptomatic patients coming for routine oncologic 18F-FDG PET/CT during the COVID-19 outbreak and found that about 11% of patients had chest findings suggestive of COVID-19 pneumonia and confirmed their findings by RT-PCR.

In our study, the CT characteristics were consistent with that published in the literature [13–18]. In COVID-19 pneumonia, the most common chest CT findings were ground-glass opacities followed by consolidation. The most common site of distribution was the lung periphery. The interlobular septal thickening was often seen. Crazy paving and reverse halo signs were characteristic of COVID 19 pneumonia; however, they were uncommon. Also, enlarged mediastinal lymph nodes and pleural effusion were rare to be encountered.

The multifocal affection was characteristic of COVID-19 pneumonia in multiple ground-glass and consolidation patches seen scattered in multiple lobes. This feature helps in the diagnosis of COVID-19 pneumonia rather than bacterial pneumonia [13, 19, 20]. The most common affected lobes were the lower lobes, mainly the dorsal segments. This agrees with Wu et al. [21], who found that the most commonly affected lung segments were the lower lobes’ dorsal and basal segments.

The reported negative initial CT rate in patients with positive COVID-19 PCR reached 17–28% [7, 22]. A study by Sabri et al. [18] found that normal CT interpreted as negative for COVID-19 in 26.1% of RT-PCR proved COVID-19 cases. Similar findings were found in our study, as negative CT was encountered in 26.8% of patients. The relatively low negative predictive value suggests that CT may not be valuable as a screening test for COVID-19, at least in earlier disease stages [23].

We found that older age is a significant predictor of death. This is in agreement with the widely known high death rate in the elderly [24, 25]. Laboratory tests usually reveal normal or reduced counts of peripheral blood leukocytes and lymphocytes at the early stages of the disease. Most patients have increased C-reactive protein and D-dimer levels [26]. In the present study, leucocytic and lymphocytic counts and, more significantly, D-dimer predict death in COVID-19 patients. D-dimer test is a good negative test with high sensitivity that can rule out pulmonary embolism in patients with low clinical risk [27]. However, it has very low specificity, especially in the intensive care unit (ICU) patients, as many other conditions are associated with raised D-dimer level [28]. Recent guidelines recommend non-contrast CT chest for only in-patient symptomatic patients for specific indications, e.g., assessing disease severity and follow-up [29]. In suspected cases, CT pulmonary angiography (CTPA) should be done as it is considered the gold standard for the diagnosis of pulmonary embolism with sensitivity and specificity of 92% and 98%, respectively [30].

This study has several limitations. First, given the small sample size, further studies with a larger sample size are still recommended. Second, it was retrospective. Further prospective cohort studies could better assess the risk factors and avoid the selection bias. Third, the current results were not correlated with the duration of illness. Further investigations are needed to clarify the time-related course of COVID-19 pneumonia.

## Conclusion

CT helps in the diagnosis and assessment of the severity of pulmonary affection of COVID 19 pneumonia. Poor clinical outcome is related to high CT severity score, high D-dimer level and low absolute lymphocytic count.

## Data Availability

The data that support the findings of this study are available from Radiology Department, Assiut University, but there are restrictions apply to the availability of data, which are used under license for this study, and so were not publicly available. Data were available from authors upon request with permission of the head of the Radiology Department, Assiut University.

## Abbreviations

COVID-19: Coronavirus disease 2019
GGO: Ground-glass opacities
RT-PCR: The real-time reverse transcriptase-polymerase chain reaction
SARS: Severe acute respiratory syndrome
OR: Odds ratio
CI: Confidence interval
